# Validation of PRISM (Pictorial Representation of Illness and Self Measure) as a novel visual assessment tool for the burden of suffering in tinnitus patients

**DOI:** 10.1186/s12955-016-0454-2

**Published:** 2016-03-22

**Authors:** Nicole Peter, Tobias Kleinjung, Lukas Horat, Sabine Schmidt-Weitmann, Martin Meyer, Stefan Büchi, Steffi Weidt

**Affiliations:** Department of Otorhinolaryngology, University Hospital Zurich, Zurich, Switzerland; Clinical Telemedicine, University Hospital Zurich, Zurich, Switzerland; Psychological Institute, University of Zurich, Zurich, Switzerland; Clinic for Psychotherapy and Psychosomatics “Hohenegg”, Meilen, Switzerland; Department of Psychiatry and Psychotherapy, University Hospital Zurich, Zurich, Switzerland

**Keywords:** Tinnitus, Quality of life, Depression, Pictorial Representation of Illness and Self Measure (PRISM), Tinnitus Questionnaire, Tinnitus Handicap Inventory

## Abstract

**Background:**

Chronic subjective tinnitus is a frequent condition that affects the subject’s quality of life. The lack of objective measures of tinnitus necessitates the use of self-reporting and often time-consuming questionnaires for evaluating tinnitus severity. The Pictorial Representation of Illness and Self Measure (PRISM) is a two dimensional pictorial method to assess the burden of suffering. Patients illustrate their burden of suffering by the distance from a “self” to an illness circle, whereby a shorter distance indicates a higher burden of suffering. The aim of this prospective observational study was to validate the burden of suffering measured with PRISM in tinnitus patients by comparing it with different standardized questionnaires currently used in tinnitus evaluation.

**Methods:**

A total of 188 patients filled out an online-based survey including sociodemographic variables and the following questionnaires: Tinnitus Handicap Inventory (THI), Tinnitus Questionnaire (TQ), WHO Quality of Life-Questionnaire (WHOQOL-BREF), and the Beck Depression Inventory (BDI). The subtle differences in the burden of suffering were accessed by using PRISM as an iPad version. Based on PRISM performance patients could easily be assigned in three groups, these being mildly, moderately, or severely affected akin to the standard questionnaires.

**Results:**

The burden of suffering measured with PRISM correlated with the tinnitus severity (THI and TQ), depressive symptoms (BDI), and health related quality of life (WHOQOL-BREF) (all *p* ≤ 0.001). In the three PRISM groups tinnitus severity (THI and TQ), and depressive symptoms (BDI) differed significantly (all *p* ≤ 0.01).

**Conclusion:**

PRISM is an easily understood and time saving method for the assessment of burden of suffering in tinnitus patients. In daily clinical practice PRISM can help to identify patients with decompensated tinnitus that require more intensive treatment.

## Background

Tinnitus is an auditory perception of sound in the absence of any external or internal acoustic stimulus. Chronic subjective tinnitus is a frequent condition with a prevalence that rates between 2.4 and 20 % [1]. A study done by the Clinical Telemedicine Department of the University Hospital Zurich (which has provided an online consultation service on health issues in all areas of medicine since 1999) shows that requests concerning tinnitus belong to the 50 most frequently raised topics [2]. Furthermore tinnitus can be associated with the impairment of health related quality of life (HRQoL) [3, 4] or depressive symptoms [5–7]. The extent to which tinnitus affects the quality of life or is linked with depressive symptoms is highly variable [7, 8]. Recent studies demonstrated an association between various subjective aspects of tinnitus, HRQoL and depressive symptoms [8, 9].

The lack of objective means of measuring tinnitus necessitates the use of self-report questionnaires for the evaluation of tinnitus severity. Several questionnaires have been developed to assess tinnitus severity or tinnitus-related impairment [8]. The *Tinnitus Handicap Inventory* (THI) is the most standardized questionnaire for assessing the tinnitus related handicap [10]. The validated German version [11] of the *Tinnitus Questionnaire* (TQ) [12] is a widely used measure of tinnitus-related psychological and psychosomatic distress.

As described in our previous study [9], the assessment of tinnitus severity has a particular relevance in clinical evaluation as it can help in the identification of patients who are at risk of developing psychological distress or depressive symptoms. At present, tinnitus questionnaires are widely used as measuring instruments in tinnitus research and in specialized tinnitus clinics. However, according to our own experience, in clinical practice most general practitioners, otologists and other clinicians do not use these questionnaires in a daily routine. A reason for this might be the time consuming aspect involved in the filling out and analyzing of these questionnaires. To address this time consuming aspect shortened forms of the THI have been developed and validated in different languages [13, 14]. Furthermore, problems in the understanding of the questions by foreign language speaking patients may complicate the assessment.

Other methods for assessing the impact of a disease are graphic tools such as pictures, charts and graphs. With a simple two-dimensional visual instrument such as the *Pictorial Representation of Illness and Self-Measure* (PRISM), the burden of suffering caused by an illness can be quickly and easily measured [15]. Burden of suffering is defined as ‘a state of severe distress associated with events that threaten the intactness of the person’ [16]. So far, PRISM has been validated as a reliable method for assessing the burden of suffering in different psychological and physical conditions such as dizziness [17], post-traumatic stress disorder [18], rheumatoid arthritis [19], dermatological diseases [20–23], and orofacial pain [24]. There is a strong correlation between the patient’s self-perceived severity of illness and their perception of the burden of suffering caused by the illness, as measured with PRISM [21–23]. Overall, the burden of suffering measured with PRISM seems to reflect an individual’s well-being in the context of illness [25]. Therefore, PRISM seems to be a reliable, feasible and useful tool in the assessment of illness [25].

To our knowledge, no previous studies have focused on the burden of suffering as assessed by the PRISM instrument in patients with tinnitus. In a prospective observational study we aimed to investigate whether PRISM can be used for this purpose. Therefore, we compared the burden of suffering, measured with the electronic version of PRISM on an iPad, with different, conventional means of measuring tinnitus severity, HRQoL and depressive symptoms. Furthermore, we aimed to investigate which sociodemographic factors contributed to the burden of suffering in tinnitus patients.

## Methods

### Participants

This study was a prospective, non-interventional, observational trial in patients suffering from tinnitus. The study was approved by the ethical committee of the Canton of Zurich and it was registered on clinicaltrial.gov (NCT01837368). All participants gave their written electronic consent.

**Fig. 1:**
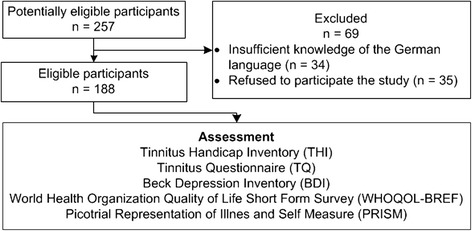
Flow diagram. From the 257 patients 188 subjects could be included in the study. Thirty-four patients were not included due to insufficient knowledge of the German language. Thirty-five patients declined to take part in the study. The assessment contained the standard questionnaires (THI, TQ, WHOQOL-BREF, and BDI) and PRISM

From December 2012 until May 2014, 257 patients (96 women (37.4 %), 161 men (62.6 %)) suffering from tinnitus were asked to participate in the study before or at their scheduled appointment at the department of Otorhinolaryngology of the University Hospital of Zurich. They were asked to complete an online survey within a time frame of two weeks before to two weeks after their consultation. Patients were included in the study when they were at least 18 years old, were fluent in the German language, had enough computer knowledge to participate in an online survey, and reported having tinnitus for a minimum of one month. From the 257 patients 188 subjects filled in the survey and completed the PRISM within the above-mentioned period of time. Thirty-four patients were not included due to insufficient knowledge of the German language. Thirty-five patients declined to take part in the study (Fig. 1).

### Assessment

The *Tinnitus Handicap Inventory* (THI) [10] is the most standardized questionnaire for assessing the tinnitus-related handicap. In the online survey of this study we used the validated German version [26] with 25 questions. There are three response options for every item: yes (4 points), sometimes (2 points), and no (0 points). Consequently, the THI total score ranges from zero to 100 and indicates the subject’s overall handicap based on their tinnitus. This total score can be categorized into five levels of tinnitus severity: slight (0 to 16), mild (18 to 36), moderate (38 to 56), severe (58 to 76), and catastrophic (78 to 100) [8]. Additionally, the 25 items of the THI can be divided into three subscales: functional, emotional and catastrophic.

The *Tinnitus Questionnaire* (TQ) [12] is a measure of tinnitus-related psychological and psychosomatic distress. The validated German version is widely used [11]. The sum score was calculated according to the validation of the TQ in the German language, which was based on 40 out of all 52 items and item numbers 5 and 20 were counted twice [8, 11]. Each item has three response alternatives: true (2 points), partly true (1 point), or not true (0 points). Therefore, the total score ranges from 0 to 84 and can be divided into four distress levels: mild (0 to 30), moderate (31 to 46), severe (47 to 59), and very severe (60–84). Furthermore, the items can be grouped into different subscales: emotional and cognitive distress, intrusiveness, auditory perceptual difficulties, sleep disturbances, and associated somatic complaints.

The THI and TQ normally reveal solid and reliable intercorrelations, meaning that they cannot be considered as independent measurements.

The *Beck Depression Inventory* (BDI) [27] was used to assess depressive symptoms. The scale consists of 21 items with four response options on a scale from 0 to 3. The total score ranges from 0 to 63 and has shown good psychometric properties [28]. This score can be categorized into the four categories of depression: minimal (0 to 9), mild (10 to 18), moderate (19 to 29), and severe (30 to 63).

The *World Health Organization Quality of Life Short Form Survey* (WHOQOL-BREF), an internationally used and well validated instrument, was used to measure the patient’s health related quality of life (HRQoL) [29]. The WHOQOL-BREF comprises 26 items assessing the following broad domains: physical health, psychological health, social relationships, environment, and global quality of life. In all domains a higher score represents a better HRQoL. The WHOQOL-BREF has been validated in different representative samples. Reference norms from the general population (*N* = 2055) are available for the German version [30].

**Fig. 2:**
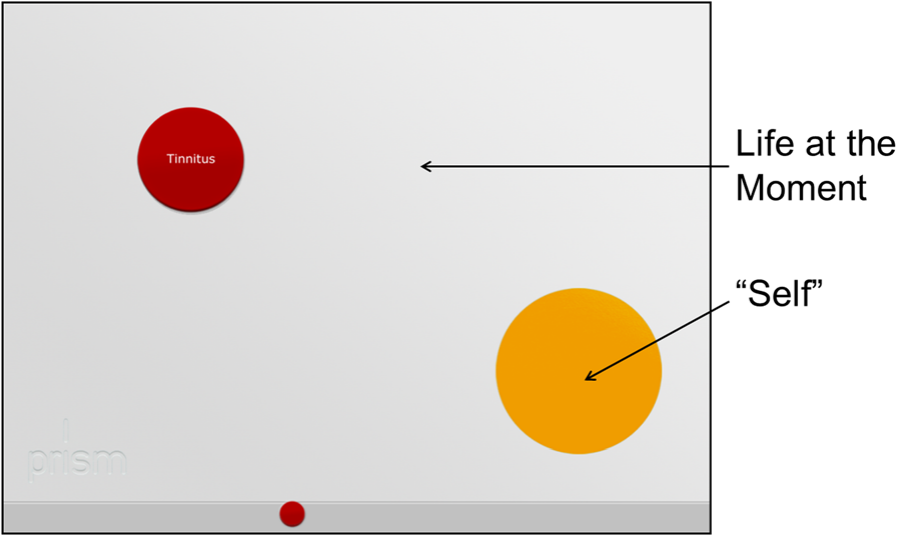
PRISM. The white panel is represented the subject’s life at that moment in time. The yellow circle at the bottom right-hand corner represented the “self”. Subjects were asked to place the red circle representing their tinnitus in relation to the yellow circle

The *Pictorial Representation of Illness and Self Measure (PRISM)* is a visual instrument to assess and quantify the burden of suffering caused by illness [15, 31, 32]. It has been validated for a variety of chronic diseases [17–24, 33]. In contrast to previous studies, PRISM was performed through an electronic version on an iPad (the application can be downloaded from www.prismium.ch). In all other aspects, the task was applied as described elsewhere [15, 20, 21, 32]. In order to permit a comparison with previous studies, all formats were normalized from the display of the iPad (147 × 196 mm) to a DIN-A4 format (210 × 297 mm). The following descriptions are based on the DIN-A4 format. The instruction was read off a standardized text while simultaneously being demonstrated on the iPad. Patients were shown a white panel (210 × 297 mm) which took up the whole display of the iPad, with a yellow circle (diameter of 70 mm) at the bottom right-hand corner (Fig. 2). Patients were asked to imagine that the panel represented their life at this moment in time, with the yellow circle representing “themselves”. Next, they were asked to imagine that another smaller red circle (diameter of 50 mm) stood for their tinnitus and were asked the following question: “Where would you place the tinnitus-circle in your life at the moment?”. To aid the comprehension of the task, an example with a blue “work-circle” was given to the patients. Individuals who define themselves through work would place the blue work-circle inside or near the yellow self-circle. Other individuals who work to earn their living but define themselves through e.g. family and hobbies would put the blue work-circle far away from the yellow self-circle. After seeing this example, patients placed the red circle representing their tinnitus in the previously described panel. The distance from the centers of the two circles derived from the PRISM was the self-illness-separation (SIS). This parameter ranges from 0 to 256 mm.

**Fig. 3:**
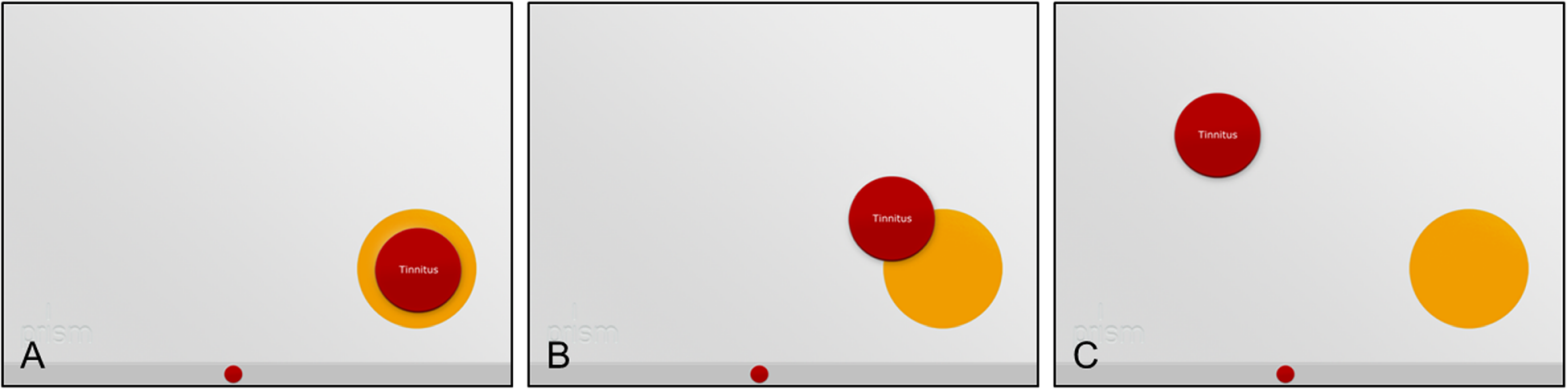
The three different PRISM groups. **a**: Group 1 with red tinnitus-circle completely inside the yellow self-circle (most severe). **b**: Group 2 with red tinnitus-circle overlapping the yellow self-circle. **c**: Group 3 with red tinnitus-circle outside the yellow self-circle (least severe)

Similar to a recent study in orofacial pain patients [24], SIS was divided into three groups reflecting the severity of the burden of suffering. The reason for turning a continuous parameter into a categorical one is that it could make it easier for the investigator to interpret the results in a short time. In group 1 the red tinnitus-circle was completely placed inside the yellow self-circle (most severe) with a SIS smaller than or equal to 10 mm; in group 2 the red tinnitus-circle was overlapping the yellow self-circle (and was not part of group 1) with a SIS ranging from 11 to 60 mm; in group 3 the red tinnitus-circle was placed outside the yellow self-circle (least severe) with a SIS bigger than 60 mm (Fig. 3).

Additionally, we accessed the time from the beginning of the explanation until the point that the patients placed the red tinnitus-circle on the white panel.

Furthermore, all patients underwent a systematic otological and audiological examination including pure tone audiometry and tinnitus matching.

### Statistical analysis

Based on previous studies about PRISM [17–24] the sample size was set on at least 180 patients. For each instrument of measurement, descriptive statistics were calculated and illustrated as means with standard deviations or as percentages. The correlations between SIS and clinical characteristics (THI, TQ, BDI and WHOQOL-BREF) were analyzed using Pearson correlation coefficients. To correlate age or duration of tinnitus with SIS, a Spearman’s correlation coefficient was applied. For different nominal variables, such as gender, inter-group comparisons of SIS were performed using Student’s *t*-test or one-way analysis of variance (ANOVA). To discriminate the three PRISM groups from each other, the mean differences of SIS, THI, TQ, BDI and WHOQOL-BREF obtained from the three PRISM groups were compared by using one-way ANOVA. To deal with multiple testing, a Bonferroni correction was applied. The level of significance was set at p ≤ 0.05 (two sided), unless otherwise specified (e.g., adjusting for multiple testing). For statistical analyses SPSS (Statistical Packages for Social Sciences, version 22.0, SPSS Inc., Chicago, IL, USA) was used.

## Results

### Study population

A total of 188 subjects were included. The mean age of participants was 46.7 years (±13.98 SD, range 18–87 years), 122 (64.9 %) were male, and the mean duration of tinnitus was 69 months (±112.1 SD, range 1–720 months). The mean SIS was 93 mm (±77 mm SD, range 1–256 mm) and the mean duration of explaining and executing PRISM was 1 min and 55 s (SD ±47 s., range 28 s. – 4 min.).

The global and psychological WHOQOL-BREF showed a significantly lower mean of the global and psychological HRQoL when compared with a German norm population [30] (Student’s *t*-test global WHOQOL-BREF: t = −4.9, df = 187, *p* < 0.0001, Student’s *t*-test psychological WHOQOL-BREF: t = −4.1, df = 187, *p* < 0.0001).

Further socio-demographic and clinical characteristics are presented in Table 1.

**Table 1:** Demographic and clinical characteristics of 188 patients with tinnitus

	Mean	SD
Age, years	46.7	18.98
PRISM, SIS in mm	93	77
THI total score (0–100)	43.7	23.5
TQ total score (0–84)	33.8	17.8
BDI total score (0–63)	9.0	6.8
WHOQOL-BREF global score	60.0	21.0
WHOQOL-BREF psych. sub score	66.6	15.8
Duration of tinnitus, month	69	112.1
	Number of patients	%
Gender, female/male	66/122	35.1/64.9
Partnership, yes/no	120/68	63.8/36.2
Education		
No degree or basic school education	16	8.5
Apprenticeship or high school diploma	75	39.8
University degree	97	51.6
Characteristics of tinnitus		
Permanent tinnitus, yes/no	144/44	76.6/23.4
Kind of tinnitus, tone/other	128/60	68.1/31.9
Increased tinnitus due to stress, yes/no	131/57	69.7/30.3

*PRISM* Pictorial Representation of Illness and Self Measure, *SIS* Self-Illness-Separation, *THI* Tinnitus Handicap Inventory, *TQ* Tinnitus Questionnaire, *BDI* Beck Depression Inventory, *WHOQOL-BREF global* WHO Quality of Life - BREF global score, *WHOQOL-BREF psych* WHO Quality of Life - BREF psychological sub score, *Partnership* married and not married relationship

### Correlation between PRISM (SIS) and clinical characteristics

SIS was significantly inversely correlated with the THI, TQ and BDI total score (all p ≤ 0.001). Furthermore, SIS significantly positively correlated with WHOQOL-BREF global score and psychological sub score (all p ≤ 0.001). Also THI, TQ, BDI, WHOQOL-BREF were all significantly associated with each other (all p ≤ 0.001). There was no significant correlation between SIS and the duration of tinnitus (Spearman’s rho = 0.089, *p* = 0.225) or age (*r* = −0.029, *p* = 0.692) (Table 2).

**Table 2:** Correlations between SIS and clinical characteristics

	PRISM; SIS	THI	TQ	BDI	WHOQOL-BREF global
PRISM; SIS	1				
THI	-.568***	1			
TQ	-.598***	.873***	1		
BDI	-.389***	.735***	.730***	1	
WHOQOL-BREF global	.371***	-.605***	-.559***	-.616***	1
WHOQOL-BREF psych.	.422***	-.633***	-.594***	-.725***	.721***
DoT^a^	.089	-.115	-.066	-.024	.127
Age^a^	-.029	-.110	.043	.022	.017

*PRISM* Pictorial Representation of Illness and Self Measure, *SIS* Self-Illness-Separation, *THI* Tinnitus Handicap Inventory, *TQ* Tinnitus Questionnaire, *BDI* Beck Depression Inventory, *WHOQOL-BREF global* WHO Quality of Life - BREF global score, *WHOQOL-BREF psych* WHO Quality of Life - BREF psychological sub score, *DoT* duration of tinnitus

Pearson correlations unless otherwise specified; ^a^Spearman’s correlation coefficient; ***p ≤ 0.001 (2-tailed)

### Association between PRISM (SIS) and socio-demographic characteristics

There were no differences in mean SIS related to the gender. An association between SIS and partnership status could be demonstrated. Subjects having a relationship had a significantly higher mean SIS than those living alone (p ≤ 0.05). There was also an association between SIS and the level of education. The mean SIS of subjects with no degree or basic school education was significantly lower than that of those with a university degree (*p* < 0.05) (Table 3).

**Table 3:** Association between SIS and socioeconomic characteristics

Condition	Mean in mm (SD)	t^1^; (F)^2^	df^1^, (df1,df2)^2^	*p*
Gender^a^		−0.70	186	0.48
Female	88 (79)			
Male	96 (77)			
Partnership^a^		−2.35	161.89	0.02
Yes	102 (82)*			
No	77 (67)*			
Education^b^		(3.45)	(2, 185)	0.034
No degree or basic school education	47 (64)*			
Apprenticeship or high school diploma	93 (77)			
University degree	101 (78)*			

*SIS* Self-Illness-Separation, *SD* standard deviation, *Partnership* married and not married relationship

*p < 0.05

^a^Student’s *t*-test

^b^one-way ANOVA

### Discrimination of the three PRISM Groups from each other

The means of the clinical characteristics for each PRISM group can be seen in Table 4. The means of the three PRISM groups in SIS, THI, TQ and BDI were significantly (at least p ≤ 0.01, mostly p ≤ 0.001) different from each other (Table 5). In the WHOQOL-BREF there was no significant difference in the global and psychological quality of life between the group 1, which positioned the red tinnitus-circle in the yellow self-circle, and the group 2, which placed the red tinnitus-circle overlapping the yellow self-circle. But the means in the global and psychological WHOQOL-BREF of these two groups were significantly different from group 3 (all p ≤ 0.001), which placed the red tinnitus-circle outside the yellow self-circle.

**Table 4:** Mean and standard deviation of the clinical characteristics in the three PRISM groups

	PRISM group 1	PRISM group 2	PRISM group 3
	(*n* = 25, 13.3 %)	(*n* = 72, 38.3 %)	(*n* = 91, 48.4 %)
SIS (mm)	3.5 (2.5)	39.0 (11.9)	160.6 (56.5)
THI	67.12 (18.62)	49.42 (20.79)	32.80 (20.47)
TQ	53.76 (12.62)	38.23 (15.10)	24.80 (15.18)
BDI	14.56 (9.43)	10.07 (6.15)	6.64 (5.20)
WHOQOL-BREF global	46.50 (20.90)	56.25 (22.40)	66.76 (17.30)
WHOQOL-BREF psych.	55.17 (16.46)	63.02 (14.87)	72.62 (15.76)

*PRISM* Pictorial Representation of Illness and Self Measure, *PRISM group 1* tinnitus-circle inside self-circle, *PRISM group 2* tinnitus-circle overlapping self-circle, *PRISM group 3* tinnitus-circle outside self-circle, *SIS* Self-Illness-Separation, *THI* Tinnitus Handicap Inventory, *TQ* Tinnitus Questionnaire, *BDI* Beck Depression Inventory, *WHOQOL-BREF global* WHO Quality of Life - BREF global score, *WHOQOL-BREF psych* WHO Quality of Life - BREF psychological sub score

**Table 5:** Multiple comparisons of the clinical characteristics in the three PRISM groups using ANOVA

	Mean differences of the PRISM groups			F (2,185)	Sig.
	Group 1/Group 2	Group 1/Group 3	Group 2/Group 3		
SIS (mm)	−35.4***	−157.1***	−121.7***	256.52	0.000
THI	17.70**	34.33***	16.63***	32.43	0.000
TQ	15.53***	28.97***	13.44***	42.58	0.000
BDI	4.49**	7.92***	3.43**	17.39	0.000
WHOQOL-BREF global	−9.75	−20.26***	−10.51**	12.32	0.000
WHOQOL-BREF psych.	−7.85	−17.45***	−9.60***	17.79	0.000

*PRISM* Pictorial Representation of Illness and Self Measure, *PRISM group 1* tinnitus-circle inside self-circle, *PRISM group 2* tinnitus-circle overlapping self-circle, *PRISM group 3* tinnitus-circle outside self-circle, *SIS* Self-Illness-Separation, *THI* Tinnitus Handicap Inventory, *TQ* Tinnitus Questionnaire, *BDI* Beck Depression Inventory, *WHOQOL-BREF global* WHO Quality of Life - BREF global score, *WHOQOL-BREF psych* WHO Quality of Life - BREF psychological sub score

**p ≤ 0.01, ***p ≤ 0.001; Bonferroni-corrected threshold for significance: 0.0027

## Discussion

The current study aimed at investigating the association between the burden of suffering measured with PRISM and standardized tinnitus questionnaires. A high burden of suffering was anticipated to be significantly correlated with a high tinnitus severity. As anticipated, a high burden of suffering (low SIS) was significantly correlated with a high tinnitus severity (high THI and TQ).

In addition to the investigation of the correlation between the burden of suffering and tinnitus severity, we divided the results of the burden of suffering (SIS) into three different PRISM groups (representing the different levels of tinnitus severity) to make it easier for the investigator to interpret the results in a short time. We then compared the means of THI and TQ between these groups. We could show significantly different means of THI between PRISM groups 1, 2 and 3.

Furthermore, the mean THI of PRISM groups 1, 2 and 3 classified in the corresponding grading system of THI showed a severe (group 1), moderate (group 2) and mild (group 3) tinnitus handicap, respectively. Similar to THI, the means of TQ between the three PRISM groups were significantly different from each other and each mean TQ of PRISM groups 1, 2 and 3 was classified in the corresponding grading system of TQ as severe (group 1), moderate (group 2) and slight (group 3) tinnitus, respectively. Therefore, the burden of suffering from tinnitus measured with PRISM is not only significantly inversely correlated with the tinnitus questionnaires, it could also be divided into three significantly different PRISM groups. Furthermore, the mean scores of THI and TQ of the three different PRISM groups are classified in different grades in the corresponding grading system of THI and TQ.

As previously stated in other studies [8, 9] we could also demonstrate a significant association of tinnitus severity (THI and TQ) with depressive symptoms (BDI) and the global and psychological HRQoL (WHOQOL-BREF). Additionally, significant inverse correlations were found between the burden of suffering from tinnitus (low SIS), depressive symptoms (high BDI), and global and psychological HRQoL (WHOQOL-BREF). Moreover, we could demonstrate that our entire study population had a significantly lower mean of global and psychological HRQoL (WHOQOL-BREF) when compared with a German norm population [30]. We can, therefore, state that suffering from tinnitus influences the HRQoL of our study population to a significant degree. However, we cannot say whether low subjective HRQoL may have fostered symptoms of tinnitus or vice-versa.

Although PRISM was performed with an electronic version on an iPad instead of the paper or conventional magnetic board version, the association between burden of suffering (SIS) with tinnitus questionnaires (THI and TQ), depressive symptoms (BDI) and HRQoL (WHOQOL-BREF) is consistent with other studies on PRISM reflecting a subjective perception of an illness [17–24, 33–35].

The present findings also suggest that the burden of suffering is not gender-related although a higher percentage of males sought medical advice concerning tinnitus symptoms (62.6 %) and took part in our survey (64.9 %). This observation is confirmed by other studies which also reported a higher prevalence for males perceiving tinnitus than for females [36, 37]. The explanation for this phenomenon is the higher degree of hearing loss amongst males, especially after the age of 50, which is most likely due to occupational noise exposure [38]. A difference to previous studies on PRISM [17, 21], is that some socioeconomic variables played a role in the burden of suffering in patients with tinnitus. The burden of suffering was significantly lower (higher SIS) for patients in a partnership compared with patients living outside a partnership. Furthermore, patients with a university degree had a significantly lower burden of suffering (higher SIS) compared to patients with no degree or basic school education. This is in line with another previous study which demonstrated a negative association of tinnitus with the degree of education and the number of household members [37, 39]. Indirectly, this finding could be an indicator for the better coping strategies of patients who have a partner or a higher education. The burden of suffering was not correlated with age and duration of tinnitus in the current sample.

Our data illustrated that the burden of suffering measured with PRISM showed a significant correlation with the conventionally used tinnitus questionnaires. This outcome is consistent with our expectations and previously published studies on PRISM [17, 31]. Consequently, PRISM as a multi-factorial construct is reliably assessing the burden of suffering in patients with tinnitus, as well as those illnesses already investigated [17–24, 33, 34]. Similar to a recent study [24], the PRISM results were divided into three different PRISM groups in order to give a direct impression of the burden of suffering in tinnitus patients. For this type of analysis the SIS is not important, as a simple optical discrimination of the relationship between one circle and the other is sufficient for the result (inside, overlapping and outside). These groups were significantly discriminant between the different levels of tinnitus severity. Notably, PRISM reliably distinguished between patients with mild compensated (group 3) versus severe, probably decompensated tinnitus (group 1). Taking into account that the mean duration of explanation and the execution of PRISM was less than two minutes, PRISM therefore may be a useful screening tool for use in routine medical consultations proposing a high burden of suffering, as an indicator for a decompensated chronic tinnitus situation. To qualify PRISM as screening tool further studies should address this issue.

At this point, we want to acknowledge the limitations of our study. Firstly, the patient population suffering from tinnitus was partly preselected as, for the most part, the patients were sent to our clinic after having been seen by a general practitioner. Secondly, the majority of the patients did not perform PRISM and the online survey on the same day, as we accepted a timeframe of two weeks between them for completion. Additionally, 13 % of the tinnitus patients in our initial population were non-German speakers and had to be excluded due to the study design. Furthermore, the answers on PRISM as a multi-factorial construct are generally influenced by other uncontrollable factors like coping strategies or personal resilience. Finally, all patients with tinnitus presumably having different underlying origin of tinnitus were included in our study. In most cases the underlying origin of tinnitus was unknown and it is therefore possible that different pathologies may have affected the PRISM results.

In conclusion, PRISM cannot completely replace the standardized tinnitus questionnaires because those provide more specific information related to different aspects like emotional distress, auditory perceptual difficulties and sleep disturbances caused by tinnitus [11]. However, our study demonstrated that PRISM is a simple approach that gives a global impression of burden of suffering in patients with tinnitus and is easily applicable in tinnitus patients in clinical contexts. It should also have great value in assessing treatment associated changes. Further to this, it might also act as an instrument for evaluating the effectiveness of treatment as demonstrated in other conditions [19, 21–23]. Future research studies should attempt to quantify the effects of tinnitus therapy by considering the change of SIS.

## Conclusion

The current study showed that the burden of suffering measured with PRISM is significantly correlated with a high tinnitus severity as measured with the standardized tinnitus questionnaires THI and TQ. Furthermore, we could demonstrate a correlation of the burden of suffering with influencing factors in tinnitus perception, such as HRQoL and depression. According to the location of the red tinnitus-circle on PRISM, we divided the answers into three different PRISM groups (inside, overlapping and outside) and could show a significantly different level of tinnitus severity as measured with THI and TQ in these three groups. Moreover, the mean scores of THI and TQ of the three different PRISM groups showed an analogous classification when compared with the corresponding grading system of THI and TQ. PRISM can be easily executed in a timesaving manner in routine medial consultations. It can facilitate the quick and simple identification of patients with decompensated chronic tinnitus, in which case further examination is recommended. Whether therapeutic effects can be illustrated with PRISM will be addressed in further studies.
